# Olfactory Dysfunction and Its Relationship with Clinical Symptoms of Alzheimer Disease

**DOI:** 10.14336/AD.2018.0819

**Published:** 2018-12-04

**Authors:** Qiujin Yu, Peng Guo, Danning Li, Lijun Zuo, Tenghong Lian, Shuyang Yu, Yang Hu, Li Liu, Zhao Jin, Ruidan Wang, Yingshan Piao, Lixia Li, Xiaomin Wang, Wei Zhang

**Affiliations:** ^1^Department of Neurology, Beijing Tiantan Hospital, Capital Medical University, Beijing, 100050, China.; ^2^Department of Geriatrics, Beijing Tiantan Hospital, Capital Medical University, Beijing, 100050, China.; ^3^Department of Physiology, Capital Medical University, Beijing, 100069, China.; ^4^Key Laboratory for Neurodegenerative Disorders of the Ministry of Education, Capital Medical University, Beijing, 100069, China.; ^5^Center of Parkinson Disease, Beijing Institute for Brain Disorders, Beijing, 100069, China.; ^6^Beijing Key Laboratory on Parkinson Disease, Beijing, 100053, China.; ^7^China National Clinical Research Center for Neurological Diseases, Beijing, 100050, China

**Keywords:** Alzheimer disease, olfactory dysfunction, self-report, Hyposmia Rating Scale, Sniffin’ Sticks test, clinical features

## Abstract

Our study aimed to analyse the olfactory dysfunction (OD) evaluations between self-report, the Hyposmia Rating Scale (HRS) and the Sniffin’ Sticks test, and the relationship between OD and clinical features of AD. Sixty patients with AD dementia, 37 patients with mild cognitive impairment (MCI) due to AD and 30 healthy controls were consecutively recruited. Olfactory function was evaluated by self-report, HRS and Sniffin’ Sticks test. Patients were divided into AD with OD (AD-OD) and AD with no OD (AD-NOD) groups based on the results of the Sniffin’ Sticks test. Cognitive symptoms and neuropsychiatric symptoms were assessed by corresponding scales, and activities of daily living (ADL) were assessed by the ADL scale. In the control, MCI due to AD and AD dementia groups, the frequency of OD was 10.0%, 13.5% and 18.3%, respectively, by self-report; 6.7%, 24.3% and 48.3%, respectively, by HRS; and 3.3%, 13.5% and 65.0%, respectively, by the Sniffin’ Sticks test. Compared to the results of the Sniffin’ Sticks test, the diagnostic coincidence rates of OD by HRS in patients with MCI due to AD and AD dementia were 89.2% and 66.7%, respectively. Compared to the AD-NOD group, the scores of global cognition and memory, visuospatial ability and attention were all decreased (P<0.05), the apathy score was increased (P<0.05), and the ADL score was elevated (P<0.01). The frequency and accuracy of OD by self-report is relatively low. HRS can be used for screening olfaction in patients with MCI due to AD. The Sniffin’ Sticks test can be used for validating OD in AD patients. AD-OD patients have severe impairments in global cognition and multiple cognitive domains of memory, visuospatial ability and attention, as well as neuropsychiatric symptoms of apathy, and thus have seriously compromised ADL.

Alzheimer disease (AD) is a progressive neuro-degenerative disease and is the commonest form of dementia in the elderly population. The incidence and prevalence of AD are increasing with the rapid aging of the population. Great attention has been paid to cognitive impairment, neuropsychiatric symptoms and activities of daily living (ADL) for AD patients. In recent years, it was found that olfactory dysfunction (OD) occurred in AD patients, even in those who were at an early stage of disease [1]. OD might predict the progression of AD [2]. However, OD has been largely ignored by both doctors and patients despite its early appearance in AD [3].

OD includes disturbances in olfactory threshold, discrimination, and identification. The majority of studies have only examined olfactory identification in patients with mild cognitive impairment (MCI) due to AD [4] or dementia due to AD (AD dementia) [5]. In a few studies, olfactory functions other than identification have been examined. Only a handful of them included more than one olfactory test, and most often these were olfactory threshold and identification [6]. However, in addition to patients with MCI due to AD and patients with AD dementia, some proportion of the normal population has OD. We therefore conducted the present study to evaluate three aspects of olfactory function, including olfactory threshold, discrimination, and identification, in control, MCI due to AD and AD dementia groups.

OD is largely ignored by patients, so we first asked each subject whether he or she had OD according to their perception of odor. Then, we used the Hyposmia Rating Scale (HRS), which might offer a simple and time-saving approach to screening OD. OD can be validated by objective and reliable tests, such as the University of Pennsylvania Smell Identification Test (UPSIT) [7] and Sniffin’ Sticks test [8]. However, UPSIT is just used to assess olfactory identification, whereas the Sniffin’ Sticks test comprehensively evaluates olfactory threshold, discrimination, and identification. We therefore determined the frequency of OD and investigated the relationship between OD and clinical symptoms of AD by the Sniffin’ Sticks test in the present study.

Studies about AD with OD (AD-OD) are limited. Most of them have focused on the manifestations of AD-OD [9] and the prediction of the conversion of MCI to AD [10,11]. However, there are few studies on the relationship between OD and clinical symptoms of AD, including cognitive impairment, neuropsychiatric symptoms and ADL. A previous study found that olfactory identification worsened as the AD deteriorated, indicating its potential as a clinical marker of AD progression [2]. However, data from another study did not support this point of view [10]. Olfactory discrimination is related to executive function and semantic memory [12], but there has been no investigation of the correlation of AD-OD with other cognitive domains, such as attention, language and visuospatial ability. Moreover, few studies on the relationship between AD-OD and neuropsychiatric symptoms, such as apathy, anxiety, depression and agitation, have been conducted.

In this study, in patients with MCI due to AD, patients with AD dementia, and control subjects, OD was investigated by self-report, HRS and the Sniffin’ Sticks test to evaluate the efficacy and clinical application of the three approaches of OD evaluation. Clinical features of AD-OD were investigated by analyzing demographic variables and the scores of rating scales for clinical symptoms of cognition, neuropsychiatric symptoms and ADL with the aim of determining the relationship between OD and AD.

## MATRIALS AND METHODS

### Ethics statement

This study met the guidelines of Helsinki Declaration on ethical principles for medical research involving human subjects, and the protocol was approved by the ethical review board of Beijing Tiantan Hospital. All participants signed written informed consents before they were recruited in the study. All methods were performed in accordance with the relevant guidelines and regulations.

### Patients with AD

Inclusion criteria: This study included patients with MCI due to AD [13] and patients with AD dementia [14] according to National Institute of Aging and Alzheimer's Association (NIA-AA) criteria.

Exclusion criteria: (1) acute respiratory infections within 3 weeks; (2) chronic nasitis and sinusitis, and chronic obstructive pulmonary disease; (3) long-term or significant exposure to volatile substances, such as pesticides, herbicides, metallic dusts, acid fumes, industrial solvents, cleaning products or sawdust; (4) severe head trauma, nasal surgery; (5) smoking and drug abuse; (6) other neuropsychiatric disorders affecting olfactory function, such as Parkinson disease, multiple sclerosis and epilepsy.

Total 97 AD patients were consecutively recruited from the Departments of Geriatrics and Neurology, Beijing Tiantan Hospital, Capital Medical University, from November 2014 to March 2017. Of the 97 AD patients, 37 were diagnosed with MCI due to AD and 60 had AD dementia.

### Control participants

Thirty healthy controls from the community were recruited based on the following criteria: (1) no cognitive impairment; (2) no intracranial diseases, including encephalitis, meningitis, cerebrovascular disease, epilepsy and tumors; (3) no psychosis or dysarthria that affected expression; (4) no acute respiratory infections within the previous 3 weeks; (5) no chronic nasitis or sinusitis or chronic obstructive pulmonary disease; (6) no long-term or significant exposure to volatile substances, such as pesticides, herbicides, metallic dusts, acid fumes, industrial solvents, cleaning products and sawdust; (7) no severe head trauma or nasal surgery; (8) no cigarette smoking or drug abuse; (9) no other neuropsychiatric disorders affecting olfactory function, such as Parkinson disease, multiple sclerosis and epilepsy.

### Collection of demographic information

Demographic variables, including gender, age, age of onset, disease duration, education level, and smoking were recorded for all AD participants. Demographic variables, including gender, age, education level, and smoking were recorded for control participants.

### Evaluation of olfactory function by Sniffin’ Sticks test

Olfactory function was evaluated for each participant by the Sniffin’ Sticks test. Sniffin’ Sticks were purchased from Burghart Messtenik Company (Tinsdaler Weg 175 22880 Wedel Germany; product number: LA-13-00005). There were a total 112 sticks, in which 48 were for testing olfactory threshold (THR), 48 for testing olfactory discrimination (DIS) and 16 for testing olfactory identification (ID).

Participants were exposed to n-butanol samples from the lowest to the highest concentration. In the olfactory THR test, the score of THR was the ranking of the Sniffin’ Sticks when participants could identify the minimal concentration of n-butanol. The lower the THR score, the worse the function of recognition.

In the olfactory DIS test, participants were instructed to name the target odor that was different from the other two odors. The score of DIS was the number of Sniffin’ Sticks that participants correctly answered. The lower the DIS score, the worse the function of discrimination.

In the olfactory ID test, participants were required to choose the name of the odor he or she smelled from 4 given choices. The score of ID was the number of Sniffin’ Sticks that participants correctly answered. The lower the ID score, the worse the function of identification.

Overall olfactory function was assessed by summing up the scores of THR, DIS and ID, which was abbreviated as TDI.

Olfactory function was identified by the following criteria deriving from a cross-sectional study of olfactory function in 3282 people by the Sniffin’ Sticks test adjusted for sex and gender [8]. OD was diagnosed in males aged 36-55 years with TDI score ≤24 points, in females aged 36-55 years with TDI score ≤28 points, and in males or females aged >55 years, with TDI score ≤19 points. No OD was diagnosed in males aged 36-55 years with TDI score ≥25 points, in females aged 36-55 years with TDI score ≥29 points, or in males or females aged >55 years with TDI score ≥20 points.

### Screening of olfactory function by HRS

HRS was validated by the Sniffin’ Sticks test in PD patients by Millar in 2012 [15] and was used to evaluate olfactory function in AD patients and control participants in this study. HRS includes 6 items, each one describing the level of olfactory function from 0 to 4 point (s), for a total score of 24 points. Total HRS score was obtained by summing up the score of each item. The higher the score of HRS, the better the function of olfaction. The optimal cut-off value for HRS was 22.5 points, with a sensitivity of 70% and a specificity of 85% [15]. Thus, AD patients with total HRS score ≥23 points and ≤22 points were defined as with OD and with no OD, respectively [15].

The sensitivity, specificity, positive predictive value and negative predictive value of HRS were calculated with reference to the results of the Sniffin’ Sticks test. Sensitivity = true positive/(true positive + false negative) x 100%, specificity = true negative/(true negative + false positive) x 100%, positive predictive value = true positive/(true positive + false positive) x 100%, negative predictive value = true negative/(true negative + false negative) x 100%, and diagnostic accordance rate = (true positive + true negative)/total x 100%. Sensitivity reflects the ability to diagnose OD by HRS. Specificity reflects the ability to judge people who actually have NOD by HRS. Positive predictive value refers to how many people diagnosed with OD truly have OD. Negative predictive value refers to how many people diagnosed with NOD truly have NOD. The diagnostic accordance rate shows the degree of agreement between HRS and the Sniffin’ Sticks test and reflects exactly the ability to diagnosis OD and NOD.

### Assessments of clinical symptoms of AD

#### Cognitive function: overall cognitive function

The Mini-Mental State Examination (MMSE) and Montreal Cognitive Assessment (MoCA) were used to rate the overall cognitive function of AD patients. Patients with illiteracy, primary education, or more than a junior education were identified as having cognitive impairment when the MMSE score was below 17, 20 or 24 points, respectively. MoCA score ≤ 26 indicated potential cognitive impairment. If the educational level of an individual was less than 12 years, 1 point was added. The lower the scores of the two scales, the severer the cognitive impairment.

#### Individual cognitive domain 

Individual cognitive domain was assessed by using a variety of rating scales, as follows:

Memory: The Auditory Verbal Learning Test [16, 17] (AVLT) was used to assess verbal memory. AVLT1-3, AVLT4 and AVLT5 stand for immediate recall, short-delayed recall and long-delayed recall, respectively. The total recall indicated by the first 5 times taking the AVLT reflects the general state of verbal memory.

The Complex Figure Test [17] (CFT)-delayed memory was used to assess visual delayed memory.

The lower the scores of the above scales, the worse the memory of the participant.

#### Visuospatial ability

CFT [17]-imitation was used to evaluate visuospatial ability. A lower score represented the worse visuospatial ability.

#### Language function

The Animal Fluency Test [18] (AFT) was used to assess language function. If the score was lower, the language function was poorer.

#### Attention

The Trail Making Test A [19] (TMT-A) was used to evaluate attention. The longer the time spent, the worse the patient’s attention was.

The Symbol Digit Modalities Test [20] (SDMT) was also used to assess attention. The lower the SDMT score, the worse the attention of the subject.

#### Executive function

The Stroop Color-Word Test [19] (SCWT) was used to evaluate executive function. The lower the score was, the worse the executive function was.

The Trail Making Test B [19] (TMT-B) was also used to assess executive function. The longer the time consumed, the worse the executive function that the patients had.

### Neuropsychiatric symptoms

#### Overall neuropsychiatric symptoms

The Neuropsychiatric Inventory (NPI) was used to assess the overall neuropsychiatric symptoms of AD patients. The higher the NPI score was, the worse the overall neuropsychiatric symptoms were.

#### Individual neuropsychiatric symptom

Individual neuropsychiatric manifestation was then assessed by using a body of rating scales:

1. Depression was assessed by the Hamilton Depression Scale (HAMD)-24 items. The higher the score was, the severer the depression was. The score >8 points suggested depression.
2. Anxiety was assessed by the Hamilton Anxiety Scale (HAMA)-14 items. The higher the score was, the severer the anxiety was. The score > 8 points indicated anxiety.
3. Agitation was assessed by the Cohen-Mansfield Agitation Inventory (CMAI). The higher the CMAI score, the worse the agitation of the participant.
4. Apathy was assessed by the Modified Apathy Estimate Scale (MAES). The higher the score was, the severer the apathy was. The score > 14 points reflected clinically meaningful apathy.

#### Activities of daily living (ADL)

The ADL scale includes basic ADL (BADL) and instrumental ADL (IADL), which were assessed by the Katz basic ADL scale [21] and the Lawton and Brody instrumental ADL scale [22], respectively. The higher the score, the worse the ADL.

**Table 1:** Demographic variables among groups of control, MCI due to AD and AD dementia.

Demographic variables	Control group (30 cases)	MCI due to AD group (37 cases)	AD dementia group (60 cases)	P1	P2
Male/total [cases/total (%)]	10/30 (33.33)	10/37 (27.03)	24/60 (40.00)	0.575	0.193
Age [years, median (quartile)]	62.00 (60.00,69.50)	64.00 (58.50, 71.00)	71.00 (62.00, 78.00)	0.529	0.019
Age of onset		60.00 (54.75, 70.00)	67.50 (58.75, 74.25)		0.022
[years, median (quartile)]					
Disease duration		3.00 (1.75, 5.00)	3.00 (1.38, 5.00)		0.559
[years, median (quartile)]					
Education [cases/total (%)]				0.098	0.846
Primary school and below	1/30 (3.33)	7/37 (18.92)	14/60 (23.33)		
Middle and high school	16/30 (53.33)	20/37 (54.05)	32/60 (53.33)		
Bachelor’s degree and above	13/30 (43.33)	10/37 (27.03)	14/60 (23.33)		
Smoking [cases/total (%)]	6/30 (20.00)	6/37 (16.22)	14/60 (23.33)	0.688	0.400

*P*1: Control group vs MCI due to AD group

*P*2: MCI due to AD group vs AD dementia group

**Table 2:** Comparisons of self-report, Hyposmia Rating Scale and Sniffin’ Sticks test in the evaluation of OD among groups of Control, MCI due to AD and AD dementia.

	Control group (30 cases)	MCI due to AD group (37 cases)	AD dementia group (60 cases)
Self-report [cases/total (%)]	3/30 (10.0)	5/37 (13.5)	11/60 (18.3)
Hyposmia Rating Scale [cases/total (%)]	2/30 (6.7)	9/37 (24.3)	29/60 (48.3)
Sniffin’ Sticks test [cases/total (%)]	1/30 (3.3)	5/37 (13.5)	39/60 (65.0)

### Data analyses

Statistical analyses were performed by SPSS Statistics 20.0 (IBM Corporation, New York, USA). *P *values < 0.05 were considered significant.

Continuous variables, if they were normally distributed, are presented as the means ± standard deviations and were compared by the two-sample t test. Continuous variables, if they were not normally distributed, are presented as median (quartile) and were compared by a nonparametric test. Discrete variables were compared by the chi-square test.

Demographic variables and the scores of olfactory functions were compared among the control, MCI due to AD and AD dementia groups.

The scores of olfactory functions, including THR, DIS, ID and TDI, and clinical symptoms, including cognitive symptoms, neuropsychiatric symptoms and ADL as determined by the corresponding rating scales, were compared between AD-OD and AD-NOD groups.

Spearman correlation analyses were performed between the scores of THR, DIS, ID, TDI and the scores of cognitive symptoms, neuropsychiatric symptoms and ADL in the AD group.

**Table 3:** Comparisons of olfactory function among groups of Control, MCI due to AD and AD dementia by Sniffin’ Sticks test.

Olfactory variables	Control group (30 cases)	MCI due to AD group (37 cases)	AD dementia group (60 cases)	*P*1	*P*2	Adjusted *P*2
TDI [points, median (quartile)]	29.50 (24.75, 31.00)	26.00 (22.00, 29.00)	17.00 (12.00, 22.00)	0.000	0.000	0.000
THR [points, median (quartile)]	6.00 (5.75, 7.00)	6.00 (4.00, 6.00)	4.00 (2.00, 6.00)	0.000	0.000	0.012
DIS [points, median (quartile)] ID [points, median (quartile)]	11.50 (8.75, 12.00) 12.00 (10.00, 13.00)	10.00 (8.00, 11.00) 10.00 (8.00, 13.00)	6.50 (4.00, 8.00) 7.00 (5.00, 9.00)	0.001 0.055	0.000 0.000	0.000 0.000

HRS: Hyposmia Rating Scale, TDI: Threshold + Discrimination + Identification, THR: Threshold, DIS: Discrimination, ID: Identification, *P*1: Control group vs MCI due to AD group, *P*2: MCI due to AD group vs AD dementia group

## RESULTS

### Comparisons of demographic variables among control, MCI due to AD and AD dementia groups

First, demographic variables, including gender, age, educational level, and smoking rate, were compared between the control group and the MCI due to AD group (Table 1). No significant differences in demographic variables were found between the two groups (*P*>0.05).

Second, demographic variables were compared between the MCI due to AD group and the AD dementia group (Table 1). The age and age of onset in the AD dementia group were significantly older than those in the MCI due to AD group (*P*<0.05). No significant difference was observed in other demographic variables between the two groups (*P*>0.05).

### Comparisons of self-report, HRS and Sniffin’ Sticks test in the evaluation of OD among control, MCI due to AD and AD dementia groups

#### (1)Self-report

Each subject was asked to report whether he or she had hyposmia. The frequency of OD in the control, MCI due to AD and AD dementia groups by self-report was 10.0%, 13.5% and 18.3%, respectively (Table 2).

Among the 30 normal controls, 3 (10.0%) reported OD, but none had OD by the Sniffin’ Sticks test.

Of the 37 patients with MCI due to AD, 5 (13.5%) reported OD, among which only 1 (20.0%) had OD and 4 (80.0%) did not by the Sniffin’ Sticks test.

**Table 4:** Comparison of Olfactory function between AD-NOD and AD-OD groups.

Olfactory variables	AD-NOD group (53 cases)	AD-OD group (44 cases)	*P*
TDI [points, median (quartile)]	25.00 (22.00, 28.50)	14.00 (10.25, 17.00)	0.000^**^
THR [points, median (quartile)]	6.00 (4.00, 6.00)	3.00 (1.00, 5.00)	0.000^**^
DIS [points, median (quartile)] ID [points, median (quartile)]	10.00 (8.00, 11.00) 10.00 (9.00, 12.50)	5.00 (3.00, 7.00) 6.00(3.00, 8.00)	0.000^**^ 0.000^**^

TDI: Threshold + Discrimination + Identification, THR: Threshold, DIS: Discrimination, ID: Identification

Of the 60 patients with AD dementia, 11 (18.3%) reported OD, among which 8 (72.7%) had OD and 3 (27.3%) did not by the Sniffin’ Sticks test.

#### (2)HRS

HRS is a scale for screening olfactory function. In this study, the frequency of OD in the control, MCI due to AD and AD dementia groups by HRS was 6.7%, 24.3% and 48.3%, respectively (Table 2).

Of the 30 subjects in the normal control group, 2 (6.7%) had OD by HRS, but nobody was demonstrated to have OD by the Sniffin’ Sticks test.

Of the 37 patients with MCI due to AD, 9 (24.3%) had OD by HRS, but only 5 (13.5%) had OD by the Sniffin’ Sticks test. Of the remaining 28 patients (75.7%) who did not present OD by HRS, nobody had OD by the Sniffin’ Sticks test. Taking the Sniffin’ Sticks test as the reference, the sensitivity, specificity, positive predictive value and negative predictive value of HRS were 100.0%, 87.5%, 55.6% and 100.0%, respectively. The diagnostic coincidence rate of OD by HRS in patients with MCI due to AD was 89.2%.

**Table 5:** Comparison of cognitive function between AD-NOD and AD-OD groups.

Cognitive variables	AD-NOD group (53 cases)	AD-OD group (4 cases)	*P*
**Global cognitive function**			
MMSE [points, median (quartile)]	25.00 (23.00, 27.50)	18.00 (11.00, 24.00)	0.000**
MoCA [points, median (quartile)]	21.00 (16.00, 25.00)	14.00 (7.00, 19.00)	0.000**
**Memory**			
AVLT-long delayed recall	3.00 (0.00, 5.75)	0.00 (0.00, 0.25)	0.001**
[points, median (quartile)]			
AVLT- the first 5 times total recall	16.50 (10.00, 26.75)	10.00 (8.00, 13.25)	0.001**
[points, median (quartile)]			
CFT- delayed memory	5.50 (0.00, 10.00)	0.00 (0.00, 2.00)	0.001**
[points, median (quartile)]			
**Language**			
AFT (points, mean ± SD)	15.54 ± 4.61	10.64 ± 4.20	0.151
**Attention**			
SDMT	22.00 (18.00, 32.00)	16.00 (8.25, 23.00)	0.009**
[points, median (quartile)]			0.000**
TMT-A-time consuming	78.00 (60.00, 108.00)	142.50 (83.25,221.00)	
[seconds, median (quartile)]			
**Visual spatial function**			0.002**
CFT- imitation	28.00 (18.00, 34.00)	8.00 (2.00, 28.00)	
[points, median (quartile)]			
**Executive function**			
SCWT- time consuming	94.00 (72.50, 120.25)	127.00 (76.50,176.75)	0.059
[seconds, median (quartile)]			
SCWT- correct number	48.00 (43.75, 50.00)	44.00 (39.25, 50.00)	0.124
[points, median (quartile)]			
TMT-B- time consuming	238.00 (195.00,240.00)	240.00 (167.00, 240.00)	0.695
[seconds, median (quartile)]			

**: *P*<0.01. MMSE: Mini-Mental State Examination, MoCA: Montreal Cognitive Assessment, AVLT: Auditory Verbal Learning Test CFT: Complex Figure Test, AFT: Animal Fluency Test, SDMT: Symbol Digit Modalities Test, TMT: Trial Making Test, SCWT: Stroop Color Word Test

Of the 60 patients with AD dementia, 29 (48.3%) had OD by HRS, 24 (40.0%) were demonstrated to have OD by the Sniffin’ Sticks test. In the remaining 31 patients (51.7%) who did not display OD by HRS, 15 had OD by the Sniffin’ Sticks test. Compared with the results from the Sniffin’ Sticks test, the sensitivity, specificity, positive predictive value and negative predictive value of HRS were 61.5%, 76.2%, 82.8% and 51.6%, respectively. The diagnostic coincidence rate of OD by HRS in patients with AD dementia was 66.7%.

#### (3)Sniffin’ Sticks test

The Sniffin’ Sticks test was adopted to objectively and comprehensively evaluate olfactory function. The frequency of OD in the control, MCI due to AD and AD dementia groups by the Sniffin’ Sticks test was 3.3%, 13.5% and 65.0%, respectively (Table 2).

The frequency of AD-OD was determined by the results of the Sniffin’ Sticks test. Of the 97 total AD patients, 44 (45.4%) were demonstrated to have OD by the Sniffin’ Sticks test. Thus, the frequency of AD-OD was 45.4%.

Forty-four (45.4%) and 53 patients (54.6%) were divided into the AD with OD (AD-OD) and AD with no OD (AD-NOD) groups, respectively.

#### Comparisons of olfactory function among control, MCI due to AD and AD dementia groups

(1) Comparisons of olfactory function between control and MCI due to AD groups

Comparison of olfactory function revealed that the scores of TDI, THR and DIS in the MCI due to AD group were all lower than those in the control group (*P*<0.01) (Table 3). However, ID score was not significantly different between the two groups (*P*>0.05).

(2) Comparison of olfactory function between MCI due to AD and AD dementia groups

Comparison of olfactory function indicated that the scores of TDI, THR, DIS and ID in the AD dementia group were lower than those in the MCI due to AD group (*P*<0.01) (Table 3). After adjusting for age and age of onset, the scores of TDI, THR, DIS and ID in the AD dementia group were still significantly lower than those in the MCI due to AD group (*P*<0.05) (Table 3).

#### Clinical evaluation of olfactory function between AD-OD and AD-NOD groups

First, in the 97 AD patients, the median scores of TDI, THR, DIS and ID were 21.00 (15.00, 26.00), 4.00 (3.00, 6.00), 8.00 (5.00, 10.00) and 8.00 (6.00, 11.00) points, respectively. Further comparison revealed that the median scores of TDI, THR, DIS and ID in the AD-OD group were all significantly lower than those of the AD-NOD group (*P*<0.01) (Table 4).

#### Relationships between olfactory function and clinical symptoms of AD

(1) Relationships between the scores of olfactory function and cognitive symptoms

MMSE score in the AD-OD group was decreased compared with the AD-NOD group (*P*<0.01) (Table 5). MMSE score was positively correlated with the scores of TDI, THR, DIS and ID (*P*<0.05) (Supplemental table 1).

MoCA score in the AD-OD group was reduced compared with the AD-NOD group (*P*<0.01) (Table 5). MoCA score was positively correlated with the scores of TDI, THR, DIS and ID (*P*<0.05) (Supplemental table 1).

The score of AVLT-delayed memory in the AD-OD group was decreased compared with the AD-NOD group (*P*<0.01) (Table 5). AVLT-delayed memory score was positively correlated with the scores of TDI, THR, DIS and ID (*P*<0.05) (Supplemental table 2).

The score of total AVLT in the AD-OD group was reduced compared with the AD-NOD group (*P*<0.01) (Table 5). Total AVLT score was positively correlated with the scores of TDI, THR, DIS and ID (*P*<0.05) (Supplemental table 2).

The score of CFT-delayed in the AD-OD group was decreased compared with the AD-NOD group (*P*<0.01) (Table 5). CFT-delayed score was positively correlated with the scores of TDI, THR and DIS (*P*<0.01) (Supplemental table 2).

There was no difference in AFT score between the AD-OD and AD-NOD groups (*P*>0.05) (Table 5).

The score of SDMT in the AD-OD group was significantly lower than that in the AD-NOD group (*P*<0.01) (Table 5). SDMT score was positively correlated with the scores of TDI, DIS and ID (*P*<0.05) (Supplemental table 3).

The time used for TMT-A in the AD-OD group was significantly prolonged compared with the AD-NOD group (*P*<0.01) (Table 5). Time spend for TMT-A was negatively correlated with the scores of TDI, DIS and ID (*P*<0.01) (Supplemental table 3).

The score of CFT-imitated in the AD-OD group was lower compared with the AD-NOD group (*P*<0.01) (Table 5). CFT-imitated score was positively correlated with the scores of TDI and DIS (*P*<0.01) (Supplemental table 3).

There was no difference in the time consumed for SCWT or TMT-B or the score of SCWT between the AD-OD and AD-NOD groups (*P*>0.05) (Table 5).

**Table 6:** Comparison of neuropsychiatric symptoms between AD-NOD and AD-OD groups.

Neuropsychiatric variables	AD-NOD group (53 cases)	AD-OD group (44 cases)	*P*
NPI [points, median(quartile)]	1.00 (0.00, 2.00)	1.00 (0.00, 4.00)	0.808
HAMD [points, median(quartile)]	5.00 (2.00, 11.50)	4.00 (1.00, 12.00)	0.816
HAMA [points, median(quartile)]	4.00 (1.00, 10.50)	3.50 (1.00, 10.25)	0.807
CMAI [points, median(quartile)]	29.00 (29.00, 29.00)	29.00 (29.00, 33.00)	0.052
MAES [points, median(quartile)]	9.50 (2.00, 17.00)	16.50 (9.00, 26.00)	0.027*

*: P<0.05. NPI: Neuropsychiatric Inventory, HAMD: Hamilton Depression Scale, HAMA: Hamilton Anxiety Scale, CMAI: Cohen-Mansfield Agitation Inventory, MAES: Modified Apathy Evaluation Scale

(2) Relationships between the scores of olfactory function and neuropsychiatric symptoms of AD

MAES score in the AD-OD group was significantly elevated compared with the AD-NOD group (*P*<0.05) (Table 6). MAES score was negatively correlated with the scores of TDI, DIS and ID (*P*<0.05) (Supplemental Table 4).

CMAI score in the AD-OD group rose compared with the AD-NOD group, which difference was close to statistical significance (*P*=0.052) (Table 6).

There was no difference in NPI number, HAMD score, or HAMA score between the AD-OD group and AD-NOD group (*P*>0.05) (Table 6).

(3) The relationships between the scores of olfactory function and ADL of AD

ADL score in the AD-OD group was higher than that in the AD-NOD group (*P*<0.01). ADL score was negatively correlated with the scores of TDI, THR, DIS and ID (*P*<0.01) (Supplemental table 5).

## DISCUSSION

Olfactory function is vulnerable to multiple factors. For example, the majority of AD patients are elderly, so they are not very sensitive to the alteration of olfactory function. In addition, the decline of olfactory function may not be evident at the early stage of AD. Hence, it is easily ignored by both patients and doctors.

A number of risk factors for AD have been identified, among which age is the most obvious [23]. In this study, the AD dementia group had older age and age of onset than the MCI due to AD group (Table 1). These data suggest that aging is involved in AD dementia. In the 2011 NIA-AA diagnostic criteria [14], AD was regarded as a continuous disease process, including stages of asymptomatic preclinical AD, MCI due to AD and AD dementia. Accordingly, the age and age of onset of patients with AD dementia might be older than that of patients with MCI due to AD.

In this study, OD was evaluated by multiple approaches. First, all subjects reported OD by themselves. The data showed that the frequency of OD in the control, MCI due to AD and AD dementia groups by self-report was 10.0%, 13.5% and 18.3%, respectively (Table 2), indicating that olfactory function was increasingly impaired as cognitive function deteriorated. However, 100%, 80% and 27.3% of patients in the control, MCI due to AD and AD dementia groups, respectively, wrongly reported OD as demonstrated by the Sniffin’ Sticks test, implying a relatively low accuracy of self-report in OD evaluation, especially in the control and MCI due to AD groups. Hence, OD reported by the two groups needed further validation by using other olfactory tests. Doty. et al [3] reported that only 6% of AD patients had complaints of OD in the early stage of disease, while 90% of AD patients actually had impairment of olfactory function as demonstrated by olfactory tests. Therefore, it is necessary to detect olfactory function by using sensitive and objective tests, particularly in the early stage of AD, to more accurately identify AD patients with OD.

Second, HRS was used for screening OD. The frequency of OD also increased from the control group to the MCI due to AD group to the AD dementia group (Table 2), illustrating that olfactory function was progressively impaired as cognitive function deteriorated. Here, in the MCI due to AD group, 9 cases (24.3%) had OD by HRS. Compared with the results from the Sniffin’ Sticks test, HRS had higher sensitivity, specificity, negative predictive value and diagnostic coincidence rate. These results suggest that HRS might serve as a valuable tool of screening OD for MCI due to AD patients for its ease, convenience, reliability and time-saving. In the AD dementia group, 29 patients (48.3%) had OD by HRS. The sensitivity, specificity, negative predictive value and diagnostic coincidence rate of HRS in the AD dementia group were all lower than in the MCI due to AD group, indicating that HRS might not be suitable for AD dementia patients.

Third, the Sniffin’ Sticks test was adopted to objectively and comprehensively evaluate olfactory function. The data showed that the frequency of OD increased in the control, MCI due to AD and AD dementia groups as above (Table 2), suggesting that olfactory function was increasingly damaged as cognitive function declined. In the control group, the frequency of OD by the Sniffin’ Sticks test was lower than that by both self-report and HRS. We speculate that normal subjects might be more sensitive to olfaction, but the frequency of OD was not high when HRS was used. The frequency of OD in this group by the Sniffin’ Sticks test was the lowest because this test was more objective and comprehensive. In the MCI due to AD group, the frequency of OD by the Sniffin’ Sticks test was lower than that by HRS. However, in the AD dementia group, the frequency of OD by the Sniffin’ Sticks was higher than that by HRS and self-report, suggesting that the Sniffin’ Sticks test was more suitable for OD evaluation in patients with AD dementia.

Of the 97 AD patients, 44 = (45.4%) had OD by the Sniffin’ Sticks test, indicating that OD was a common non-cognitive symptom of AD. Comparing the above three methods of olfactory evaluation, the Sniffin’ Sticks test was able to find = more AD patients with OD, which might be attributed to its comprehensive actions of assessing olfaction in multiple aspects.

In this study, compared with the control group, olfactory threshold and discrimination of the MCI due to AD group was significantly impaired (Table 3). Compared with the MCI due to AD group, olfactory threshold, discrimination and identification of the AD dementia group were all significantly decreased (Table 3), even after adjusting age and age of onset. The above data further illustrate that the more severe the cognitive impairment, the worse the olfactory dysfunction.

In this study, the scores of TDI, THR, DIS and ID in the AD-OD group were all significantly decreased compared to the AD-NOD group (Table 4), illustrating that AD-OD was characterized by an overall decline of olfactory threshold, discrimination and identification.

The impaired cognitive domains of AD include memory, attention, language, visuospatial ability and executive function. The MMSE and MOCA scales cover the above cognitive domains and are commonly used for evaluating overall cognitive impairment. In this study, compared with the AD-NOD group, the overall cognitive function of the AD-OD group was significantly impaired, as indicated by the decreased scores of MMSE and MoCA scales (Table 5). Furthermore, the scores of the two scales were both positively correlated with the scores of TDI, THR, DIS and ID (Supplemental table 1), showing that olfactory function was increasingly impaired as cognitive level declined in AD patients.

A previous investigation reported an association between olfactory identification and cognition in AD patients by using MMSE and UPSIT for evaluation of global cognitive function and olfactory identification, respectively [2]. In the current study, in contrast to the above investigation, in addition to olfactory identification, olfactory threshold and discrimination by the Sniffin’ Sticks test were also associated with overall cognitive function as rated by MMSE and MoCA, suggesting that the Sniffin’ Sticks test has advantages for investigating overall OD and its relationship with cognitive impairment in AD patients.

In this study, AVLT and CFT were used to rate verbal memory and visual delayed memory, respectively. The AD-OD group had more seriously impaired overall memory and delayed memory than the AD-NOD group (Table 5). Moreover, the overall memory and delayed memory drastically deteriorated, as total olfactory function and olfactory threshold, identification and discrimination were all evidently impaired (Supplemental table 2). Additionally, visual delayed memory declined with compromised overall olfactory function, olfactory threshold and discrimination (Supplemental table 2). A recent meta-analysis [24] suggested that olfactory identification and discrimination were impaired in AD patients, indicating that high-level olfactory tasks involved specific cognitive processes, while olfactory threshold rather relied on low-level perceptual processes [9, 25]. In contrast to the results from other researchers reporting that olfactory identification could predict memory decline [26], the current study indicates that overall OD and impairments of olfactory threshold, identification and discrimination all might predict the decline of memory, particularly delay-memory, which might be because the Sniffin’ Sticks test evaluates olfactory function comprehensively while UPSIT, used by most of other investigators, only assesses olfactory identification.

In the present study, SMDT and TMT-A were used to assess attention, including visual scanning, attention segmentation, tracking and motion speed [20]. The AD-OD group had significantly poorer attention than the AD-NOD group (Table 5). In addition, attention was further compromised when olfactory identification and discrimination were increasingly declined (Supplemental table 3). Deficit in attention might occur at earlier stages of AD [27], and attention might be the second impaired cognitive domain followed by memory, earlier than language and visuospatial ability [28]. Olfaction and advanced cognition have common brain regions, such as the hippocampus, amygdala, temporal lobe. The brain regions activated by attention overlap with the olfaction-related ones, which might be the common anatomical basis for OD and attention deficit in AD patients [29]. The olfactory function of the AD-OD group was significantly impaired in the current investigation, and more importantly, the attention deficit of AD patients was related to olfactory identification and discrimination. Therefore, we hypothesize that AD patients have to concentrate their attention to experience and remember every odorant in the process of olfactory identification and discrimination tests. Hence, attention deficit influenced the process of identification and discrimination of AD patients.

CFT-imitation was used to evaluate visuospatial ability [17]. Our data imply that visuospatial ability in the AD-OD group was impaired compared with the AD-NOD group (Table 5). Moreover, with the impairment of visuospatial ability, the olfactory discrimination of AD patients was significantly declined (Supplemental table 3). Visuospatial impairment is one of the prominent symptoms of AD patients, and it even appears in the MCI stage [30]. Two pathways are responsible for visuospatial ability. The ventral pathway, originating from the occipital lobe and projecting to the lower temporal cortex, is mainly in charge of perception, identification of objects seen by the naked eye and the storage of spatial memory in the medial temporal lobe and hippocampus. The dorsal pathway, originating from the occipital lobe and projecting to the parietal lobe, prefrontal cortex, premotor cortex and medial temporal lobe, is mainly in charge of formation of visuospatial memory, visual navigation and subsequent processing of visuospatial ability [31]. Moreover, the olfaction-related brain regions include the medial temporal lobe and hippocampus. Therefore, we hypothesize that olfactory function and visuospatial ability share a common anatomical basis.

Apathy is a prevalent neuropsychiatric manifestation of AD, which leads to a compromised daily function and increased burden of caregivers [32]. A previous study reported a specific association between olfactory identification performance and apathy severity, suggesting that olfactory dysfunction and apathy might result from the progression of disease pathology in shared neural substrates [33]. In this study, the AD-OD group was more likely to suffer from apathy than the AD-NOD group (Table 6). With the aggravation of apathy, the function of olfactory identification and discrimination were increasingly impaired (Supplemental table 4). The apathy of AD patients was associated with olfactory discrimination. The core of apathy is a lack of motivation for emotion, cognition and behavior, which might affect the desire to participate in the tests of odor identification and discrimination. Therefore, dysfunction of olfactory discrimination of AD might be related to apathy.

The present investigation showed that the CMAI score of the AD-OD group was higher than that of the AD-NOD group, and the difference was close to statistical significance (Table 6), illustrating that the AD-OD group might be more prone to agitation than the AD-NOD group. The agitation in MCI due to AD patients and AD dementia patients is related to atrophy of frontal lobe, insular lobe, amygdala, cingulate gyrus and hippocampus [34], and coincidently, the insular lobe, amygdala and hippocampus are also olfaction-related brain regions, indicating OD and agitation share common neuroanatomical basis.

This study revealed that OD dramatically impaired the ADL of AD patients. We speculate that the damage to global cognitive function and multiple cognitive domains of memory, visuospatial ability and attention together with severer apathy amplified the negative impact on ADL of patients in the AD-OD group.

In summary, the frequency and accuracy of OD by self-report is relatively low. HRS can be used for screening olfaction in patients with MCI due to AD. The Sniffin’ Sticks test can be used for validating OD in AD patients. OD is prevalent in AD patients, as evidenced by the overall declines of olfactory threshold, discrimination and identification. AD-OD patients have severe impairments in global cognition and multiple cognitive domains of memory, visuospatial ability and attention, as well as neuropsychiatric symptoms of apathy, and thus have seriously compromised ADL.

## Supplementary data

Supplementary data is available online at www.aginganddisease.org/EN/10.14336/AD.2018.0819
