# Supplementary material for: Olfactory Dysfunction and Its Relationship with Clinical Symptoms of Alzheimer Disease
Source: Aging Dis. 2018 Dec 4;9(6):1084–95. doi: 10.14336/AD.2018.0819 (PMC6284764; doi:10.14336/AD.2018.0819)
Supplement: Supplementary file 1 — Supplementary data is available online at www.aginganddisease.org/EN/10.14336/AD.2018.0819 [file AD-9-6-1084-s.pdf]

SUPPLEMENTARY DATA

Olfactory Dysfunction and Its Relationship with Clinical Symptoms of Alzheimer Disease

Qiujin Yu[1], Peng Guo[2], Danning Li[1], Lijun Zuo[2], Tenghong Lian[1], Shuyang Yu[2], Yang Hu[2], Li Liu[2], Zhao Jin[1], Ruidan Wang[1], Yingshan Piao[2], Lixia Li[2], Xiaomin Wang[3,4,5,6], Wei Zhang[1,2,4,5,6,7,*]

[1]Department of Neurology, Beijing Tiantan Hospital, Capital Medical University, Beijing, 100050, China. [2]Department of Geriatrics, Beijing Tiantan Hospital, Capital Medical University, Beijing, 100050, China. [3]Department of Physiology, Capital Medical University, Beijing, 100069, China. [4]Key Laboratory for Neurodegenerative Disorders of the Ministry of Education, Capital Medical University, Beijing, 100069, China. [5]Center of Parkinson Disease, Beijing Institute for Brain Disorders, Beijing, 100069, China. [6]Beijing Key Laboratory on Parkinson Disease, Beijing, 100053, China. [7]China National Clinical Research Center for Neurological Diseases, Beijing, 100050, China

© 2018. Yu Q et al. Published online at http://www.aginganddisease.org/EN/10.14336/AD.2018.0819

SUPPLEMENTARY DATA

Supplementary Table 1. Correlation between olfactory function and global cognitive function in AD patients

	MMSE		MoCA	
	r	*P*	r	*P*
TDI	0.630	**0.000****	0.540	**0.000****
THR	0.290	**0.004****	0.227	**0.027***
DIS	0.583	**0.000****	0.490	**0.000****
ID	0.617	**0.000****	0.540	**0.000****

*：P＜0.05, **：P＜0.01

Abbreviations: **TDI**=Threshold+Discrimination+Identification; **THR**=Threshold; **DIS**= Discrimination; **ID**= Identification; **MMSE** = Mini-Mental State Examination; **MoCA** = Montreal Cognitive Assessment.

Supplementary Table 2. Correlation between olfactory function and memory in AD patients.

	AVLT- long delayed recall		AVLT- the first 5 times total recall		CFT- delayed memory	
	r	*P*	r	*P*	r	*P*
TDI	0.418	**0.000****	0.462	**0.000****	0.387	**0.002****
THR	0.239	**0.027***	0.248	**0.021***	0.345	**0.007****
DIS	0.334	**0.002****	0.377	**0.000****	0.392	**0.002****
ID	0.411	**0.000****	0.458	**0.000****	0.180	0.168

*：P＜0.05, **：P＜0.01

Abbreviations: **TDI**=Threshold+Discrimination+Identification; **THR**=Threshold; **DIS**= Discrimination; **ID**= Identification; **AVLT** = Auditory Verbal Learning Test; **CFT** = Complex Figure Test.

Supplementary Table 3. Correlation between olfactory function and attention and visual spatial function in AD patients.

	SDMT		TMT-A		CFT-imitation	
	r	*P*	r	*P*	r	*P*
TDI	0.308	**0.009****	-0.447	**0.000****	0.342	**0.005****
THR	0.174	0.146	-0.100	0.364	0.242	0.051
DIS	0.286	**0.016***	-0.427	**0.000****	0.396	**0.001****
ID	0.264	**0.026***	-0.469	**0.000****	0.180	0.149

*：P＜0.05, **：P＜0.01

Abbreviations: **TDI**=Threshold+Discrimination+Identification; **THR**=Threshold; **DIS**= Discrimination; **ID**= Identification; **SDMT** = Symbol Digit Modalities Test; **TMT** = Trial Making Test; **CFT** = Complex Figure Test.

© 2018. Yu Q et al. Published online at http://www.aginganddisease.org/EN/10.14336/AD.2018.0819

SUPPLEMENTARY DATA

Supplementary Table 4. Correlation between olfactory function and MAES in AD patients.

	r	*P*
TDI	-0.249	**0.016***
THR	-0.171	0.103
DIS	-0.225	**0.031***
ID	-0.210	**0.044***

*：$P < 0.05$

Abbreviations: **TDI**=Threshold+Discrimination+Identification; **THR**=Threshold; **DIS**= Discrimination; **ID**= Identification; **MAES** = Modified Apathy Evaluation Scale.

Supplementary Table 5. Correlation between olfactory function and ADL in AD patients.

	r	*P*
TDI	-0.513	**0.010***
THR	-0.274	**0.009****
DIS	-0.487	**0.000****
ID	-0.468	**0.000****

*：$P < 0.05$, **：$P < 0.01$

Abbreviations: **TDI**=Threshold+Discrimination+Identification; **THR**=Threshold; **DIS**= Discrimination; **ID**= Identification; **ADL**= Activity of Daily Living

© 2018. Yu Q et al. Published online at http://www.aginganddisease.org/EN/10.14336/AD.2018.0819
